# Applying artificial intelligence technology to assist with breast cancer diagnosis and prognosis prediction

**DOI:** 10.3389/fonc.2022.980793

**Published:** 2022-08-31

**Authors:** Meredith A. Jones, Warid Islam, Rozwat Faiz, Xuxin Chen, Bin Zheng

**Affiliations:** ^1^ School of Biomedical Engineering, University of Oklahoma, Norman, OK, United States; ^2^ School of Electrical and Computer Engineering, University of Oklahoma, Norman, OK, United States

**Keywords:** breast cancer, machine learning, deep learning, computer aided detection, computer aided diagnosis, mammography

## Abstract

Breast cancer remains the most diagnosed cancer in women. Advances in medical imaging modalities and technologies have greatly aided in the early detection of breast cancer and the decline of patient mortality rates. However, reading and interpreting breast images remains difficult due to the high heterogeneity of breast tumors and fibro-glandular tissue, which results in lower cancer detection sensitivity and specificity and large inter-reader variability. In order to help overcome these clinical challenges, researchers have made great efforts to develop computer-aided detection and/or diagnosis (CAD) schemes of breast images to provide radiologists with decision-making support tools. Recent rapid advances in high throughput data analysis methods and artificial intelligence (AI) technologies, particularly radiomics and deep learning techniques, have led to an exponential increase in the development of new AI-based models of breast images that cover a broad range of application topics. In this review paper, we focus on reviewing recent advances in better understanding the association between radiomics features and tumor microenvironment and the progress in developing new AI-based quantitative image feature analysis models in three realms of breast cancer: predicting breast cancer risk, the likelihood of tumor malignancy, and tumor response to treatment. The outlook and three major challenges of applying new AI-based models of breast images to clinical practice are also discussed. Through this review we conclude that although developing new AI-based models of breast images has achieved significant progress and promising results, several obstacles to applying these new AI-based models to clinical practice remain. Therefore, more research effort is needed in future studies.

## Introduction

The latest cancer statistics data for the USA estimates that in 2022, 31% of cancer cases detected in women are breast cancer with 43,250 cases resulting in death. This accounts for 15% of total cancer-related deaths (1). Thus, breast cancer remains the most diagnosed cancer among women with the second highest mortality rate. Over the past three decades, population-based breast cancer screening has played an important role in helping detect breast cancer in the early stage and reduce the mortality rate. From 1989 to 2017, the mortality rate of breast cancer dropped 40% which translates to 375,900 breast cancer deaths averted (2). Even though the mortality rate continues to decline, the rate of decline has slowed from 1.9% per year from 1998-2011 to 1.3% per year from 2011-2017 (2). However, the efficacy of population-based breast cancer screening is a controversial topic due to the low cancer prevalence (≤0.3%) in annual breast cancer screening resulting in a low cancer detection yield and high false-positive rate (3). This high false positive rate is indicative of a high rate of unnecessary biopsies which is not only an economic burden but also leads to unnecessary patient anxieties which often result in women being less likely to continue with routine breast cancer screening (4). Conversations pertaining to the benefits and harms of screening mammography as well as its efficacy in decreasing breast cancer mortality as screening exams do not reduce the incidence of advanced/aggressive cancers are now common (5). For example, detection of ductal carcinoma *in situ* (DCIS) or early invasive cancers that will never progress or be of risk to the patient are occurring at a disproportionately higher rate than aggressive cancers. This is referred to as overdiagnosis and often results in unnecessary treatment that may cause more harm than the cancer itself (6). Thus, improving the efficacy of breast cancer detection and/or diagnosis remains an extremely pressing global health issue (7).

While advances in medical imaging technology and progress towards better understanding the complex biological and chemical nature of breast cancer have greatly contributed to the large decline in breast cancer mortality, breast cancer is a complex and dynamic process, making cancer management a difficult journey with many hurdles along the way. The cancer detection and management pipeline has many steps, including detecting suspicious tumors, diagnosing said tumors as malignant or benign, staging the subtype and histological grade of a cancer, developing an optimal treatment plan, identifying tumor margins for surgical resections, evaluating and predicting response to chemo or radiation therapies, or predicting risk of future occurrence or reoccurrence. In this clinical pipeline, medical imaging plays a crucial role in the decision-making process for each of these tasks. Traditionally, radiologists will rely on qualitative or semi-quantitative information visually extracted from medical images to detect suspicious tumors, predict the likelihood of malignancy, and evaluate cancer prognosis. The clinically relevant information may include enhancement patterns, presence or absence of necrosis or blood, density and size of suspicious tumors, tumor boundary margin spiculation, or location of the suspicious tumor. However, interpreting and integrating information visually detected from medical images to make a final diagnostic decision is not an easy task.

Although mammography is the most frequently employed imaging modality in breast cancer screening, its performance is often unsatisfactory with lower sensitivity (i.e., missing 1 in 8 cancers during interpretation) and very high false positive rates (i.e., <30% of biopsies are malignant) (8). Thus, the downfalls of mammography have led to an increase in the use of other adjunct imaging modalities in clinical practice including ultrasound (US) and dynamic contrast enhanced magnetic resonance imaging (DCE-MRI) (9, 10). Digital breast tomosynthesis (DBT) is a newer modality that is commonly used in which X-ray images are taken over multiple angles in a limited range (i.e., ± 15° and the acquired scanning data is reconstructed into quasi-3D breast images to reduce the impact of dense breast tissue overlap in 2D mammograms (11). Additionally, several other new imaging modalities including contrast enhanced spectral mammography (CESM) (9, 10), phase contrast breast imaging (12), breast computed tomography (13), thermography and electrical impedance tomography of breast imaging (14), and molecular breast imaging (15), have also been investigated and tested in many prospective studies or clinical trials. However, using more imaging modalities for breast cancer detection and diagnosis increases the workload of radiologists in busy clinical practice. Over the last three decades, computer-aided detection and diagnosis (CAD) schemes are being rapidly developed to optimize the busy clinical workflow by assisting radiologists in more accurately and efficiently reading and interpreting multiple images from multiple sources (16, 17).

In the literature, CAD is often differentiated as computer-aided detection (CADe) or computer-aided diagnosis (CADx). The goal of CADe schemes is to reduce observational oversight by drawing the attention of radiologists to suspicious regions in an image. Commercialized CADe schemes of mammograms have been in clinical use since 1998 (18). One study reported that in 2016 CADe was used in about 92% of screening mammograms read in the United States (18, 19). Despite the wide scale clinical adoption, the utility of CADe schemes for breast cancer screening is often questioned (20–22). On the other hand, the goal of computer-aided diagnosis (CADx) schemes is to characterize a suspicious area and assign it to a specific class. US FDA approved the first CADx scheme of breast MR images, QuantX by Qlarity Imaging (Chicago, IL) in 2017 (23). The goal of QuantX is to assist radiologists in deciding if a lesion is malignant or benign by providing a probability estimation of malignancy. This software has yet to be extensively adopted and requires much more clinical testing.

Despite great research efforts and the availability of commercialized CAD tools, the added clinical value of CAD schemes and ML-based prediction models for breast images is limited. Thus, more novel research efforts are needed to explore new approaches (24). While using radiological features from medical images to infer phenotypic information has been done for many years, recent rapid advances in bioinformatics coupled with the advent of high performing computers has led to the field of radiomics. Radiomics involves the computation of quantitative image-based features that can be mined and used to predict clinical outcomes (25). In medical imaging, radiomic techniques are used to extract a large number of features from a set of medical images to quantify and characterize the size, shape, density, heterogeneity, and texture of the targeted tumors (26). Then, a statistics-based feature analysis tool such as Lasso regression or a machine learning (ML) based pipeline is applied to identify small sets of features that are more clinically relevant to the specific application. One method to ensure the extracted features contain some clinical relevance is to segment the tumor region and extract features from there. Despite the relative simplicity of extracting relevant radiomics features, automated tumor segmentation remains a major challenge. Thus, many radiomics-based schemes use manual or semi-automated tumor segmentation. Additionally, recent enthusiasm for deep learning based artificial intelligence (AI) technology has led to new approaches for developing CAD schemes which are being rapidly explored and reported in the literature (27). Several studies have compared CAD schemes using conventional radiomics and deep learning methods to investigate their advantages and limitations (28, 29). Deep learning (DL) based CAD schemes are appealing as majority of such CAD schemes eliminate the need for tedious error prone segmentation steps and no longer need to compute and select optimal radiomic features since deep learning models can extract features directly from the medical images (30). However, despite the challenge of how to achieve high scientific rigor when developing AI-based deep learning models (31), applying AI technology to develop CAD schemes has become the mainstream technique of the CAD research community. Additionally, new AI-based models are being expanded to include broad clinical applications in realms beyond cancer detection and diagnosis, such as prediction of short-term cancer risk and prognosis or clinical outcome.

In order to help researchers better understand state-of-the-art research progress and existing technical challenges, several review articles have recently been published with a variety of goals, such as a review of deep learning (DL) models developed for breast lesion detection, segmentation, and classification (27), radiomics models developed to classify breast lesions and monitor treatment efficacy (32), and how to optimally apply DL models to three commonly used breast imaging modalities (mammograms, ultrasound, and MRI) (33). The focus of this review paper is different from the previously published review articles for the following reasons. First, our paper details the recent advances in both radiomics and DL-based AI technologies to develop new prediction models. Second, this review paper does not review and discuss CADe (lesion detection or segmentation) schemes. It focuses on three more challenged application realms namely, prediction of breast cancer risk, tumor classification (diagnosis) and cancer prognosis (treatment response). Third, to help readers better understand the scientific rationales of applying new AI-based models of medical image to predict breast cancer risk, classify breast lesions, and predict cancer prognosis, this paper reviews recent studies that demonstrate the important relationship between medical image features and the tumor environment (genomic biomarkers), which supports the physiological relevance of radiomics based studies. Last, based on this review process, we are able to summarize several important conclusions that may benefit future research efforts in medical imaging of breast cancer. For this purpose, the rest of this paper is organized as follows. Section two briefly discusses the correlation of extracted medical image features and the tumor environment, followed by section three that surveys recent studies, which detail novel image-based applications of both radiomics and DL-based new AI-supported CAD schemes in three application fields. Lastly, section four discusses and summarizes key points that can be learned or observed from this review paper and future perspectives in developing CAD schemes of breast images.

## Relationship between medical image features and tumor environment

A major focus of breast cancer research in the medical imaging field is uncovering the relationships between medical image features and the tumor microenvironment to better predict clinical outcomes (**Table 1**). Since traditional CAD schemes involve handcrafting a set of features, it is important to understand what kind of descriptors correlate with cancer specific genomic biomarkers, based on radiomic concepts (25), so that optimal and descriptive handcrafted feature sets can be chosen. Additionally, if an image-based marker is widely established as a biomarker for a specific hallmark of cancer such as sustaining proliferative signaling, evading growth suppressors, invasion and metastasis, angiogenesis, or resisting cell death, then monitoring changes in that image-based marker overtime will have high degree of predictive power in many aspects of the cancer management pipeline (32).

**Table 1:** Studies of correlating image-based features with tumor physiology.

Year	Author	Imaging Modality	Image Based Features Extracted	Physiological Features	Relevant Results
2015	Li et al. (34)	DCE-MRI	Quantitative Kinetic Features: K^trans^, K_ep_, V_e_, ADC	MVD and Proliferation	K^trans^, K_ep_, and ADC closely correlate with MVD and Proliferation
2021	Xiao et al. (35)	DCE-MRI	Shape, intensity, and texture features	MVD	MVD associates with SER, WF, and radiomic features
			Semi-Quantitative Kinetic Features: PE, SER, FTV, WF		
2019	Mori et al. (36)	DCE-MRI	Semi-Quantitative Kinetic Features: IER, SER, TIE	MVD	A, α, Aα, AUC30, and TIE significantly correlate with MVD
			Quantitative Kinetic Features: EMM derived metrics: A, α, Aα, AUC30		
2016	Kim et al. (37)	DCE-MRI	Quantitative Kinetic Features: K^trans^, K_ep_, V_e_,	MVD and VEGF	MVD correlates with V_e_ and there is significant association between K^trans^, tumor size, and MVD
2014	Li et al. (38)	DCE-MRI	Semi-Quantitative Kinetic Features: longest dimension, tumor volume, SER, initialAUC	pathological response to chemotherapy	SER and K_ep_ are significantly different between responders and non-responders (p<0.05) and can be used to predict breast cancer response to NACT
			Quantitative Kinetic Features: K^trans^, K_ep_, V_e_, v_p_, and τ^i^		
2007	Yu et al. (39)	DCE-MRI	Quantitative Kinetic Features: K^trans^, K_ep_	response to chemotherapy based on RECIST	Tumor size significantly correlates with K^trans^ and K_ep_ and change in tumor size is a better response predictor than both K^trans^ or K_ep_
			Tumor size		
2020	Kang et al. (40)	DCE-MRI	Quantitative Kinetic Features: K^trans^, k_ep_, v_e_, and v_p_	ER, PR, HER2, Ki67, p53, EGFR, CK5/6 and lymphovascular space invasion	High K^trans^ and k_ep_ associate with poor prognostic histopathologic factors
2019	Braman et al. (41)	DCE-MRI	Texture and statistical features	HER2+	DCE-MRI texture and statistical features can identify molecular subtype of HER2+ breast cancer from HER2- breast cancers
2016	da Rocha et al. (42)	Mammography	Texture features from the local binary pattern of images	Malignant or benign lesion	GLCM features derived from the Local Binary Pattern have the best results for lesion classification ACC: 88.31% SEN: 85% SPE: 91.89%
2015	Zhu et al. (43)	DCE-MRI	Size, shape, morphological, enhancement texture, kinetic curves, enhancement-variance	miRNA expression, protein expression, gene mutations, transcriptional activities, and gene copy number variation	Transcriptional activities of various genetic pathways positively associate with tumor size, blurred tumor margin and irregular tumor shape, The miRNA expressions associates with the tumor size and enhancement texture
2018	Drukker et al. (44)	DCE-MRI	Semi-Quantitative Kinetic Features: Most enhancing tumor volume (METV)	recurrence free survival based on clinical examination after surgery	METV from pre-NACT and early treatment scans associate with recurrence-free survival
2006	Varela et al. (45)	Mammography	Texture features to characterize contrast and spiculations from the interior, border, and outer area of the mass	Malignant or benign lesions	Features from the mass border and outer regions contain the most information for distinguishing lesions.
2020	La Forgia et al. (46)	CESM	Statistical features	ER, PR, HER2, Ki67, Grade, Triple-negative	Statistical radiomic features extracted from CESM can predict histological outcomes
2017	Wu et al. (47)	DCE-MRI	Semi-Quantitative Kinetic features: FTV features, BPE features	molecular subtypes based on IHC	DCE-MRI based features may be able to non-invasively determine the subtype of a breast cancer
			Morphological and texture features		

SEN, sensitivity; SPE, Specificity; ACC, Overall accuracy.

For example, many studies investigated the correlation between image-based biomarkers and tumor mechanisms of angiogenesis. As tumors grow and metastasize, there is a decrease in the amount of available oxygen due an increase in demand, resulting in a hypoxic environment (33, 48–51). To adapt to the newly hypoxic environment, the tumor will enter an angiogenic state which changes the microvasculature. In this state the tumor will switch on angiogenic growth factors such as vascular endothelial growth factor (VEGF) and fibroblast growth factors (FGF) to stimulate the formation of new capillaries so that oxygen and nutrients can adequately feed the tumor (48). This process is known as angiogenesis, which is a hallmark of most cancers that can be characterized by non-hierarchical, immature, and highly permeable vasculature that looks obviously different from normal vasculature (52). Traditionally, angiogenesis is indirectly quantified as micro-vessel density (MVD) after immunohistochemical staining of tumor tissue. While high MVD has been established as a biomarker of poor prognosis and correlated with increased levels of angiogenesis, quantification of MVD is subject to inter- and intra-reader variability, making MVD a non-reproducible and non-standardized marker (53). Thus, development of a quick and non-invasive biomarker that can differentiate between highly immature angiogenic vasculature and normal vasculature has been a hot research topic over the past decade (48, 54).

DCE-MRI is a non-invasive method to detect and characterize the tumor microenvironment. Specifically, dynamic/kinetic image features computed from DCE-MRI characterize the permeability and perfusion kinetics of the tumor microvasculature which can reflect tumor angiogenesis. Many studies have been conducted to correlate quantitative and semi-quantitative DCE-MRI based kinetic features with MVD to demonstrate the relationship between DCE-MRI and tumor angiogenesis (34–37). Peak signal enhancement ratio (peak SER) and washout fraction (WF) are two semi-quantitative metrics extracted from the contrast enhancement curve that reflect the clearance of a contrast agent from the tumor. These metrics directly relate to a highly angiogenic state as rapid washout will occur with a large number of immature and leaky vessels (35). Extracting quantitative features from DCE-MRI requires a pharmacokinetic analysis which requires at high temporal resolution, often resulting in a poor spatial resolution. Clinical DCE-MRI scans prioritize spatial resolution as opposed to temporal resolution, which makes it difficult to do a fully quantitative analysis of clinical DCE-MRI scans. Most studies that have a goal of quantitative analysis of DCE imaging may not be appropriate for clinical use. However, studies have shown that quantitative DCE-MRI parameters such as, K^trans^ and K_ep_, correlated well with angiogenesis markers and can be used to predict response to treatment or risk of recurrence (34). Physiologically, K_ep_ is a marker of the efflux of contrast agent. High K_ep_ values indicate two observations of tumor microenvironment. The first indicates a strong blood flow with highly permeable vessels which represents existence of an irregular and highly vascularized space associated with tumor angiogenesis. The second indicates the smaller extravascular extracellular space, meaning large quantities of the contrast agent cannot accumulate here; this is expected as there will be an increase in cell density in the tumor environment (38). Technical details pertaining to the extraction of semi-quantitative and fully quantitative kinetic features is beyond the scope of this review, interested readers should explore the following manuscripts for more information (55, 56). While there are many studies exploring the correlations between K^trans^ and K_ep_ and cancer prognosis, there are inconsistent conclusions of the biological relevance of these markers which make studies using kinetic DCE-MRI features non-reproducible (39, 40).

**Figure 1:**
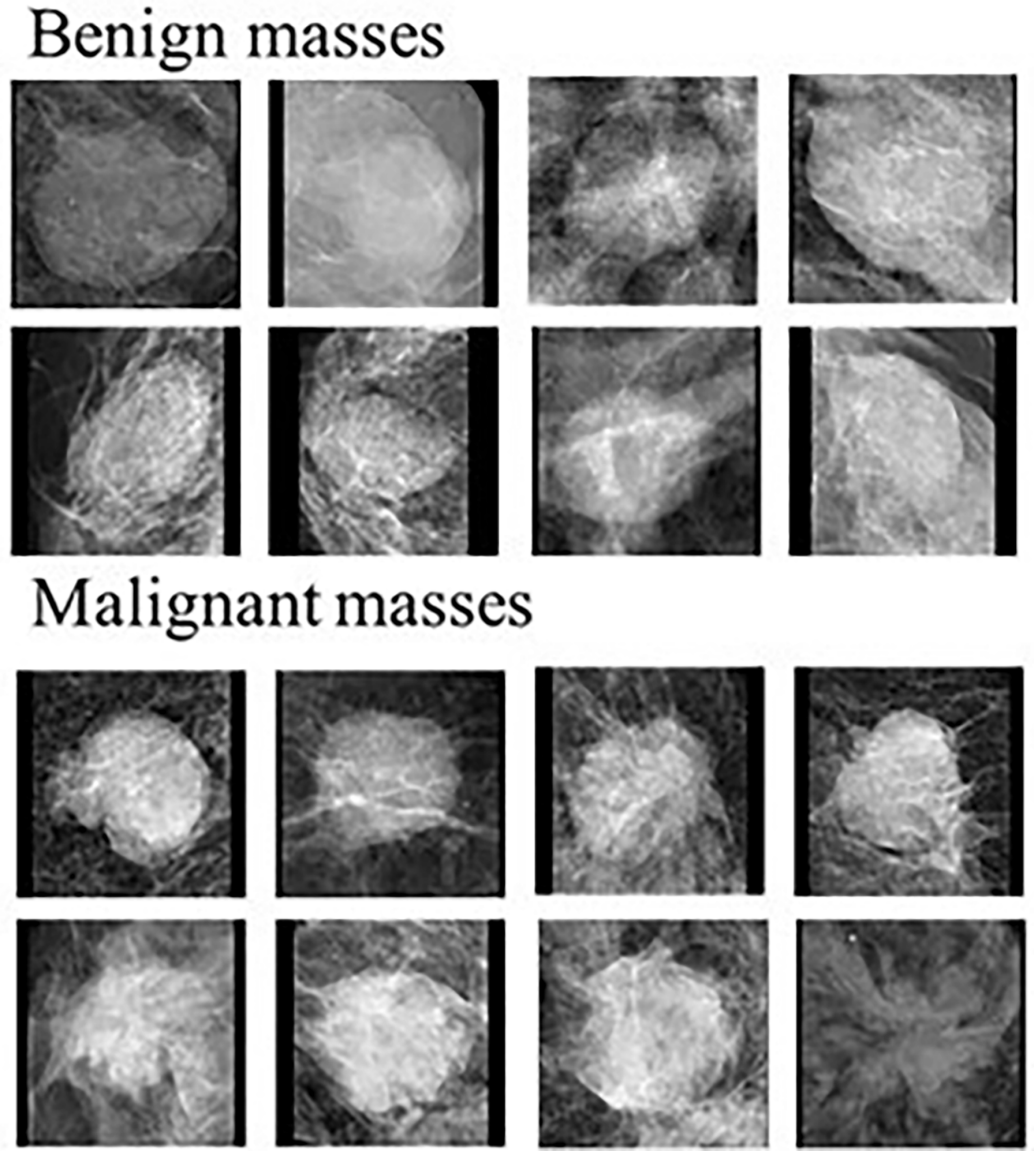
Examples of benign and malignant masses seen on mammograms. Modified from (58).

Recent studies demonstrated that radiomics features are thought to be more robust and reproducible than kinetic features computed from breast MRI for different prediction tasks (i.e., classification between malignant and benign tumors, prediction of axillary lymph node metastasis, molecular subtypes of breast cancer, tumor response to chemotherapies and overall survival of patients) (57). For example, malignant tumors as see on mammograms are typically irregular in shape with spiculated margins and architectural distortions while benign tumors are typically rounded with well-defined margins (**Figure 1**) (58–60). Quantification of these features can help train robust ML classifiers to better differentiate between benign and malignant masses. Features that describe the shape of the tumor may include eccentricity, diameter, convex area, orientation, and more. Shape based features may help differentiate between traditionally round benign tumors and spiculated malignant tumors. While shape features are important, breast compression during mammography makes extraction of these features difficult (60). Features can also be extracted to quantify the spiculations of the tumors which will be particularly helpful for detecting malignant breast tumors (45). First order statistical features are basic metrics that describe the distribution of intensities within an image, this includes mean, standard deviation, variance, entropy, uniformity, and others. For example, entropy quantifies the image histogram randomness which can quantify heterogeneity of the image patterns (61). Texture features belong to the biggest group of radiomics features, which are extremely useful for image recognition and image classification tasks (62, 63). Gray-level cooccurrence matrix (GLCM) based features and gray-level run length matrix (GLRLM) based features are two example of common texture features that characterizes the heterogeneity of intensities within a neighborhood of pixels. Quantification of the heterogeneity of tumors is one of the advantages of radiomics-generated imaging markers as heterogeneity is often very difficult for radiologists to visually capture and quantify in clinical practice.

**Figure 2:**
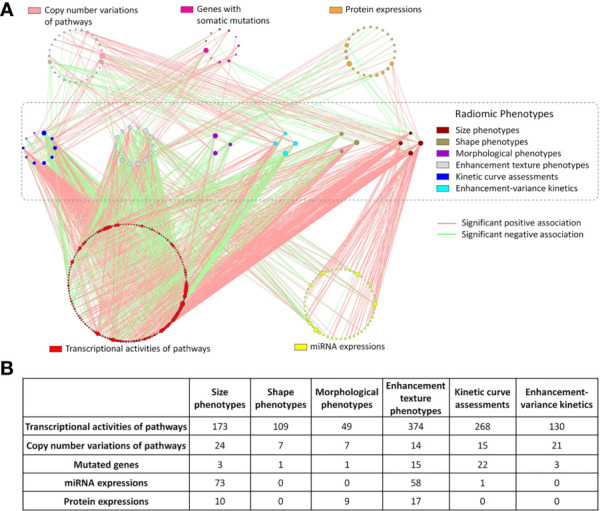
Results of mapping radiomic features extracted from DCE-MRI images of breast cancer to genomic markers. **(A)** Each line represents a statistically significant association between nodes. Each node represents either a genomic feature or radiomic phenotype. The size of the node reflects the number of connections relative to other nodes in its circle. **(B)** Displays the number of significant associated between the 6 different radiomic categories and the genomic features (43).

While identification of physical or biological reasoning for the correlations between image-based markers and cancer specific traits is lacking, there are some studies that do correlate radiomics based features with cancer specific markers that have been obtained from IHC analysis or genomic assays (35, 41). For example, Xiao et al. assessed the correlation between radiomic based DCE-MRI features with MVD in order to identify angiogenesis in breast cancer using DCE-MRI (35). GLCM and GLRLM derived textural features extracted from 3D segmented tumor regions were found to significantly correlate with MVD, therefore, correlate with angiogenesis levels. GLCM derived features from ROIs represented by the local binary patterns were also shown to be extremely useful for distinguishing malignant and benign masses detected on mammograms (42). Radiogenomics is the field that incorporates radiomics based features with patient specific genomic information. Correlation of the image-based features that characterize cancer through genetic information pertaining to tumor hormone receptors and genetic mutations can be very helpful for predicting risk of cancer recurrence and thus help develop optimal personalized treatment plans. Quantitative MRI-based features of tumor size, shape, and blood flow kinetics have been mapped to cancer specific genomic markers (**Figure 2**) (43, 44, 64). This is a great step forward in development of non-invasive techniques for understanding cancer on a molecular level.

Although DCE-MRI is an important imaging modality used to study the tumor microenvironment and predict tumor staging and/or response to therapies, other modalities have also been investigated for this purpose. For example, contrast enhanced spectral mammography (CESM) has been attracting broad clinical research interest as an alternative to DCE-MRI due to its advantages of low cost, high image resolution, and fast imaging acquisition times. Like DCE-MRI, injection of an intravenous contrast agent in CESM imaging allows for the visualization of contrast enhancement patterns which give insight into the vascular arrangement in the breast tissue. One recent paper reviewed 23 studies that investigated CESM and demonstrated that textural features and/or enhancement patterns obtained from CESM can differentiate between malignant and benign breast lesions as benign lesions often display weak and uniform contrast uptake with enhancing wash-out patterns, while malignant lesions tend to display quick decreasing wash-out patterns (65). As a result, many research studies have recently been conducted and published that compare CESM and DCE-MRI. These studies have demonstrated that CESM could achieve quite comparable performance as DCE-MRI in breast tumor diagnosis (i.e., classifying between malignant and benign tumors) (66), staging or characterizing suspicious breast lesions (46, 67), and predicting or evaluating breast tumor response to neoadjuvant therapy (68). Thus, in the last several years, exploring and extracting image features from CESM also attracts research interest in developing new quantitative image markers or CAD schemes in breast cancer research field (69).

In previous studies, radiomics features are often only extracted from the segmented tumor regions, meaning potentially valuable information of the environment surrounding the tumor and background regions is ignored. To overcome this issue and improve the accuracy of prediction models, several studies report the importance of extracting features from the targeted or global breast parenchyma as these regions may also contain important information relating to cancer state (45, 47). While there has been a wide variety of radiomics features extracted from many different locations for different cancer applications, there is no consensus on what features make up an optimal feature set. Deciding what features should be extracted remains dependent on the goal of the individual study.

## Applications of AI-based quantitative image analysis and prediction models

Rapid advances in AI technologies have promoted the development of new quantitative image feature analysis-based prediction models in breast cancer research. In addition to the conventional CADe and CADx applications, novel AI-based models have also been expanded to new applications. In this section, we review the development and applications of AI-based prediction models in three applications namely, cancer risk prediction, tumor diagnosis or classification, and cancer prognosis prediction or response to treatment (**Tables 2** **–** **4**). There exists an extremely large number of studies pertaining to AI in breast cancer in the three realms mentioned. We apply the following criteria and steps to select the most relevant studies. The titles and abstracts of potentially relevant papers in the literature database (i.e., PubMed and Google Scholar) were first analyzed for terms related to either breast cancer risk (**Table 2**), breast cancer diagnosis/classification or computer aided diagnosis of breast cancer (**Table 3**), and breast cancer treatment response or prognosis prediction (**Table 4**). Papers were then selected if a ML or a DL method was used for predictive modeling and breast image derived features or breast images were used as model inputs. Thus, all studies also use predominantly imaging data as an input to the model. Studies were omitted if there was no explicit methodology of how the model was trained and tested or if the study lacked novelty. Studies that use solely statistical methods or do not report AUC values to make predictions were also omitted from this review. All papers listed in **Tables 2**–**4** are published in the last 8 years. It should be noted that some studies investigate and report performance values for multiple combinations of features or multiple classifiers, we report only the performance results of the best model.

**Table 2:** Studies of developing AI-based image feature analysis models to predict breast cancer risk.

Year	Author	Imaging Modality	# of Images	Feature Information	ML Model	Evaluation Metrics
2018	Heidari et al. (70)	Mammography	570	43 features from the discrete cosine transform of the ROI and the spatial domain	SVM	AUC: 0.70 ± 0.04
2015	Sun et al. (71)	Mammography	340	765 texture features from multiscale subregions	SVM RBF Kernel	AUC: 0.729 ± 0.021
						PPV: 0.657 (94/140)
						NPV: 0.755 (151/200)
2018	Mirniaharikandehei et al. (72)	Mammography	1044	8 existing CADe based features	Logistic Regression	MLO based AUC: 0.65 ± 0.017
						CC based AUC: 0.586 ± 0.018
2015	Tan et al. (73)	Mammography	870	79 texture and density features	two stage ANN	AUC: 0.725 ± 0.026
2014	Gierach et al. (74)	Mammography	237	38 texture features	Bayesian ANN (BANN)	AUC: 0.72 ± 0.08
2017	Li et al. (75)	Mammography	456	4096 features from last fully connected layer of AlexNet pretrained on ImageNet	SVM	AUC: 0.83
2018	Saha et al. (76)	MRI	133	8 BPE features	multivariate logistic regression	AUC: 0.700
2019	Portnoi et al. (77)	MRI	1656	–	ResNet18 pretrained imageNet and fine tuned	AUC: 0.638 ± 0.094
2019	Yala et al. (78)	Mammography	88994	–	ResNet18	AUC: 0.70 (95% CI: 0.64, 0.73)
2021	Yala et al. (79)	Mammography	275,674	–	MIRAI	AUC: 0.76-0.79
						SEN: 26.0%-41.5%
						SPE: 85.2%-93.1%

AUC, area under ROC curve; SEN, sensitivity; SPE, Specificity; PPV, Positive predictive value; NPV, Negative predictive value.

**Table 3:** Studies of developing new CADx models to classify between malignant and benign breast tumors.

Year	Author	Imaging Modality	# of images	Feature Information	Model	Evaluation Metrics
2020	El-Sokkary et al. (80)	Mammography	322	20 Shape and Texture Features	SVM RBF Kernel	PSO Segmentation ACC: 89.5%
						GMM Segmentation ACC: 87.5%
2016	Dalmis et al. (81)	MRI	395	23 Shape and Kinetic Features	Random Forest	AUC: 0.8543
2017	Qiu et al. (82)	Mammography	560	–	8 Layer CNN	AUC: 0.790 ± 0.019
2020	Yurttakal et al. (83)	MRI	200	–	multilayer CNN	ACC: 98.33%
						SEN: 1.0
						SPE: 0.9688
2020	Hassan et al. (84)	Mammography	600	–	AlexNet pretrained on ImageNet and fine tuned	ACC: 98.29%
						SEN: 0.9782
						SPE: 0.9876
				–	GoogleNet pretrained on ImageNet and fine tuned	Acc: 95.63%
						SEN: 0.9047
						SPE: 0.9822
2019	Mendel et al. (85)	Mammography and DBT	78	VGG19 pretrained on ImageNet as a Feature Extractor	SVM	Mammography AUC: 0.810 ± 0.05
						2D DBT AUC: 0.86 ± 0.04
						Key DBT AUC: 0.89 ± 0.04
2021	Caballo et al. (86)	breast CT	284	1354 radiomic features	fusion of radiomic features and CNN based features through MLP	AUC: 0.947
2017	Antropova et al. (87)	Mammography	739	VGG19 pretrained on ImageNet as a Feature Extractor and radiomic features	fusion of radiomic features and CNN based features to a SVM RBF Kernel	AUC:0.86
		Ultrasound	2393			AUC:0.90
		MRI	690			AUC:0.89
2015	Tan et al. (88)	Mammography	1896	96 radiomic features	Multistage ANN	AUC: 0.779 ± 0.025
2019	Li et al. (89)	Mammography	182	32 lesion-based features 45 parenchymal features from contralateral breast	Bayesian ANN	AUC: 0.84 ± 0.03
2020	Heidari et al. (90)	Mammography	1000	12 Structural Similarity Index Features	SVM	AUC: 0.84 ± 0.016
						ACC: 79.00%
2020	Moon et al. (91)	Ultrasound	1687	–	Ensemble of VGGNet, ResNet, and DenseNet	ACC: 91.10%
						SEN: 85.14%
						SPE: 95.77%
						Precision: 94.03%
						F1: 89.36%
						AUC: 0.9697
			697			ACC: 94.62%
						SEN: 92.31%
						SPE: 95.60%
						Precision: 90%
						F1: 91.14%
						AUC: 0.9711

AUC, area under ROC curve; SEN, sensitivity; SPE, Specificity; ACC, Overall accuracy; F1, F1 index.

**Table 4:** Studies of developing new AI-based models to predict tumor response to chemotherapy.

Year	Author	Imaging Modality	# Of Images	Feature Information	ML Model	Evaluation Metrics
2017	Giannini et al. (92)	DCE-MRI	44	27 textural features	Bayesian Classifier	ACC: 70%
						SPE: 0.72
2015	Michoux et al. (93)	DCE-MRI	69	3 kinetic features, 2 BI-RADS based features, 21 texture- based features	Logistic Regression	ACC: 74%
						SEN: 0.74
						SPE: 0.74
					K-means clustering	ACC: 68%
						SEN: 0.84
						SPE: 0.62
2015	Aghaei et al. (94)	DCE-MRI	68	39 contrast enhanced features from both segmented malignant tumor and background parenchymal enhancement regions	ANN	AUC: 0.96 ± 0.03
						ACC: 94%
						SEN: 0.88
						SPE: 0.98
2016	Aghaei et al. (95)	DCE-MRI	151	10 global kinetic features	ANN	AUC: 0.83 ± 0.03
2018	Ravichandran et al. (96)	DCE-MRI	166	–	CNN	AUC: 0.85
						ACC: 82%

AUC, area under ROC curve; SEN, sensitivity; SPE, Specificity; ACC, Overall accuracy.

### Prediction of breast cancer risk

Women at a high risk for developing breast cancer should undergo supplemental screening exams as early detection is necessary to ensure the best prognosis (97). However, the existing risk models are mainly built based on epidemiological studies that integrate risk factors based on groups of sampled women such as: family history, hormonal and reproductive factors, breast density, obesity, smoking history, and alcohol intake, and output a breast cancer risk estimate (98, 99). By reporting odds ratios or relative risks, these risk models typically do not have discriminatory power applying to individual women. Thus, cancer detection yield in currently defined high risk groups of women remains quite low (< 3%) using mammography plus MRI screening (100). Meanwhile, up to 60% of women diagnosed with breast cancer are not considered high risk patients (101). This coupled with the increased attention to establish a new paradigm of personalized breast cancer screening highlights the need for identifying a non-invasive biomarker or developing AI-based prediction models that can better stratify women with high or low risk of developing breast cancer in the short term based on individual testing.

Since previous studies have found that women with dense breast have a higher risk of developing breast cancer (102–106), it then leads that many studies aim to quantify breast density from screening mammograms so that patients can be informed if they have dense breast therefore are at a higher risk. It is the hope that informing women of their breast density and the risks associated with dense breast will encourage supplemental and more frequent screening exams. The American College of Radiology developed the Breast Imaging Reporting and Data System (BI-RADS) to group mammographic density into one of four categories. While BI-RADS has been used extensively, it is often unreliable as the categorization varies between observers. Machine learning and deep learning techniques have been developed that quantify breast density using computerized schemes to make this a more robust metric (107–110). While many studies have shown a correlation between breast density and breast cancer risk (111–113), this metric alone is often not enough to create robust risk assessment models (102, 114). Recent studies indicate that texture-based features may have a higher discriminatory power in stratifying women based on breast cancer risk (107, 115, 116). MRI images from The Cancer Genome Atlas (TCGA) project of the National Cancer Institute (NCI) were used to demonstrate that quantitative radiomic features extracted from breast MRI images can replicate observer-rated breast density based on BI-RADS guideline (117).

In addition to the measured breast density from mammograms, other types of medical images have been explored to develop new imaging markers or AI-based prediction models to predict breast cancer risk in individual women, particularly the short-term risk, which can help better stratify women into different breast cancer screening groups (**Table 2**). Heidari et al. developed a AI-based prediction scheme to predict the risk of developing breast cancer in the short term (less than 2 years) based on features extracted from negative screening mammograms that had enhanced breast density tissue (70). The dataset used in this study included craniocaudal (CC) views of 570 negative screening mammograms that had a follow up screening exam within 2 years where 285 of these cases were then cancer positive as confirmed by tissue biopsy and 285 cases remained screening negative. The breast area was segmented from each initial negative screening mammogram and enhanced to better visualize the dense tissue as opposed to the fatty tissues. Forty-three global features were computed from the spatial domain and discrete cosine transform domain of both the left and right CC view images. This study takes advantage of the bilateral asymmetry between two breasts when creating the final feature vector that is then used to train a support vector machine (SVM) model which produces a likelihood score that the next sequential screening exam is positive. The results of this scheme were significantly better than the same scheme that does not include the segmentation and dense tissue enhancement step, emphasizing that there is important textural information in the dense tissue of negative screening mammograms that can be used to predict if there is a short-term risk of developing breast cancer.

Like conventional CADe schemes, integrating all four views of screening mammograms enables development of new cancer risk prediction models with increased performance. Mirniaharikandehei et al. investigated the hypothesis that CADe-generated false-positive lesions contain valuable information that can help predict short-term breast cancer risk (72). The motivation for this study is driven by the fact that some early abnormalities picked up on CADe schemes may have a higher risk of developing into detectable cancers in the short-term (118, 119). All cases used in this study were negative screening exams where some of these cases contained early suspicious tumors that were only considered detectable in a retrospective review of the images. A CADe scheme was applied to right and left CC and mediolateral oblique (MLO) view images and then a feature vector was created that describes the number of initial detection seeds, the number of final false positives, the average, and the sum of all detection scores. To quantify the bilateral asymmetry, the features from the left and right CC or MLO views were summed to create one CC and one MLO view feature vector with four features in each vector. Two independent multinominal logistic regression classifiers were trained, one using the CC view feature vector and another using the MLO view feature vector. The results indicated that using the MLO view model achieved higher prediction accuracy, which indicates image features computed from CC and MLO views are different since mammograms are 2D projection images and fibroglandular tissue may appear quite different along the two projection directions. Since CADe schemes are routinely used in the clinic, this study provides a unique and cost-effective approach for developing CADe generated biomarkers from negative screening exams to help predict short term breast cancer risk. Tan et al. also took advantage of all four views of the breast and the bilateral asymmetry between breasts to predict short term breast cancer risk (73). In this study, eight groups of features were extracted from either the whole breast region or the dense tissue region of the breast to train a two-stage artificial neural network (ANN). Each feature set was used independently and in combination to train the model. The best performing model was developed when the model was trained using GLRLM based texture features computed from the dense breast regions. Both studies demonstrate that using bilateral asymmetry features computed from CC and MLO views is advantageous in that overlapping dense fibroglandular tissue can be visualized in two different configurations, providing more information about the dense tissue which is a known risk factor for breast cancer development. Clinical adoption of computerized models that can predict short-term breast cancer risk will be extremely valuable to stratify women and decide optimal intervals and methods of breast cancer screening (i.e., whether need to add breast MRI to mammography).

Genetic risk factors are also measured and used by epidemiological studies to indicate the lifetime risk of developing breast cancer. One of these genetic risk factors is an autosomal dominant mutation in the BRCA1 or BRCA2 gene. Up to 72% of women who inherit the BRCA1 mutation and 69% of women who inherit the BRCA2 mutation will develop breast cancer in their lifetime (120). Many women are unaware of their BRCA1/2 status when going in for a screening mammogram. Identification of BRCA1/2 status from routine mammographic images will be clinically useful for determining high-risk individuals. Gierach et al. conducted a texture analysis study of breast cancer negative mammograms to differentiate individuals with BRCA1/2 mutations from those without a BRCA1/2 mutation based on 38 texture features extracted from the breast parenchyma on CC view mammograms (74). After performing feature selection, five features were used to train a Bayesian artificial neural network (BANN) model that outputs a likelihood of having a BRCA1/2 mutation which would classify the individual as high risk. Individuals with BRCA1/2 mutations used in this study were on average 10 years younger than the group without BRCA1/2 mutations. When an age-matched testing dataset was used to evaluate the performance of the BANN model and an AUC of 0.72 ± 0.08 was observed. Results of this study demonstrate that radiomic based texture features extracted from negative screening mammograms can help identify women who have BRCA1/2 mutations. The significance of this study highlights that image analysis of screening mammograms can be expanded to include risk stratification in addition to detection of suspicious tumors.

Breast parenchymal patterns are another biomarker that has been established as a tool for cancer risk prediction (104, 105, 116, 121). Extracting texture features from the breast parenchyma provides local descriptors that can characterize the physiological conditions of the breast tissue which may give more insight into breast cancer risk than breast density or BRCA mutation status. Li et al. used deep transfer learning with pre-trained CNNs to extract features directly from the breast parenchyma depicted on the CC view of FFDM images to differentiate between high-risk patients with a BRCA mutation and the low-risk patients and to differentiate between high-risk patients with unilateral cancer and the low-risk patients (75). In this study, regions of interest (ROIs) were selected from the central region directly behind the nipple as this region has been shown to give best results for describing breast parenchyma (116). ROIs were then input to a pretrained CNN and features were extracted from the last fully connected layer. In addition, texture-based features were also extracted from the ROIs so that the results of deep transfer learning-based classifier and traditional radiomic based classifier can be analyzed. A fusion classifier was created that used features extracted from the pretrained deep CNN and traditional texture features. The fusion classifier was able to differentiate BRCA mutation carriers from low-risk women and unilateral cancer patients from low-risk women with an AUC of 0.86 and 0.84, respectively. Additionally, the pre-trained CNN extracted features were able to differentiate between unilateral breast cancer patients and low risk patients significantly better than using traditional texture features, where AUC = 0.82 and AUC = 0.73, respectively. This study demonstrates the advantages of exploring deep learning techniques independently and in combination with conventional machine learning techniques to better stratify patients on breast cancer risk. In addition to extracting one ROI from one mammogram, other studies investigate the effect of using either multiple ROIs or global features to develop breast cancer risk assessment models. For example, Sun et al. extracted texture features from multiple subregions within the mammogram that had relatively homogeneous densities and fused the features to train an SVM with a radial basis function (RBF) kernel to predict short-term breast cancer risk (71). The classifier trained using multiscale fusion of features extracted from different density subregions showed superior performance to the classifier trained using features extracted from the whole breast. Zheng et al. developed a fully automated scheme that captures the texture of the entire breast parenchyma using a lattice-based approach (122). Using smaller local windows to extract features provided the best performance when compared to single ROI and may lead to improved model performance in predicting breast cancer risk.

Besides analyzing negative mammograms, the level of background parenchymal enhancement (BPE) on breast MRI has also demonstrated power in predicting breast cancer risk (123–125). BPE refers to the volume and intensity enhancement of normal fibroglandular tissue after intravenous contrast is injected. The hypothesis is that high levels of BPE is associated with a high risk of developing breast cancer, hence why radiologists may group women into risk groups based on BPE (126). However, there is high inter-reader variability in radiologist interpretation of BPE suggesting that developing computerized schemes to quantify BPE has the potential to produce a more robust marker to predict breast cancer risk. Saha et al. automatically quantified the BPE from screening MR exams to predict future breast cancer risk within two years using a logistic regression classifier (76). In the study, eight BPE features were extracted from the fibroglandular tissue mask from both the first post-contrast fat-saturated sequence and the T1 nonfat-saturated sequence. Five breast radiologists also reviewed MR images and categorized each case as either minimal, mild, moderate, or marked BPE according to the BI-RADS guideline. The predictive performance of the multivariate logistic regression model trained using quantitative BPE features yielded higher performance than that of the qualitative BPE assessment of the five radiologists, suggesting that computerized quantification of BPE is a more accurate predictor of breast cancer risk.

Several studies have compared new image feature analysis models with pre-existing epidemiology-based statistical models in predicting cancer risk. For example, Portnoi et al. developed a deep learning breast cancer risk prediction model using DCE-MRI taken from a high-risk population (77). The 3D MR images were converted to 2D projection images using the axial view of the maximum intensity projection (MIP) and then used to fine tune a ResNet-19 CNN that had been pretrained on the ImageNet dataset. Results from the MRI-based deep learning model were compared with the Tyrer-Cuzick model and a logistic regression model that used all risk factors from the Tyrer-Cuzick model in addition to the qualitative BPE assessment made by an expert radiologist based on the BI-RADS guidelines. The AUC of the MRI-based deep learning model, Tyrer-Cuzick model, and logistic regression model were reported as, 0.638 ± 0.094, 0.493 ± 0.092, and 0.558 ± 0.108, respectively. Study results demonstrate that new MRI-based deep learning model has higher discriminatory power to predict breast cancer risk than the existing epidemiology-based risk prediction models.

Finally, based on the hypothesis that new imaging markers and the existing epidemiology-based risk factors may contain complementary information, Yala et al. sought to combine traditional risk factors and image-based risk factors extracted from mammograms using deep learning to investigate whether fusion of the two would yield a superior 5-year risk prediction model (78). In this study, ResNet18 was trained, validated, and tested using 71,689, 8,554 and 8,869 images acquired from 31,806, 3,804 and 3,978 patients, respectively. Four different risk prediction models were compared, namely: the Tyrer-Cuzick Model, a logistic regression model using standard clinical risk factors, the deep learning model, and a hybrid model using traditional clinical risk factors and the deep learning model (AUC = 0.62,0.67,0.68, 0.70, respectively). This work laid the foundation for the development of the MIRAI model in 2021 (79), which predicts the risk of developing breast cancer for each year within the next 5 years. All four mammograms acquired in routine screening (LCC, LML, RCC, RML view) are passed as an input to this model which first go through an image encoder, next to an image aggregator, then to a risk factor predictor, followed by an additive-hazard layer. MIRAI model was first trained and validated using 210,819 and 25,644 screening mammography exams from 56,786 and 7,020 patients from Massachusetts General Hospital (MGH), respectively. MIRAI model was then tested on three different testing sets, one acquired from MGH that contained 25,855 exams from 7,005 patients, the second acquired from Karolinska University Hospital in Sweden that contained 19,328 exams from 19,328 patients, and the third acquired from Chang Gung Memorial Hospital in Taiwan that contained 13,356 exams from 13,356 patients, respectively. AUCs obtained from MIRAI model was significantly higher than those yielded by Tyrer-Cuzick model and both the hybrid deep learning model and image based deep learning model developed in 2019 foundational study (81). Thus, MIRAI model is unique for a few reasons, the first being that traditional clinical risk factors are incorporated into the imaging feature analysis model as the previous Yala et al. study (78) demonstrated that addition of this information will improve performance. If traditional risk information is not provided, MIRAI model is still able to predict cancer risk from mammographic image features. This increases its potential clinical utility in clinics that may not record many risk factors used in Tyrer-Cuzick models. Second, MIRAI model focuses directly on clinical implementation by training the model on a large dataset and validating this model on different datasets.

In summary, the above studies demonstrate that imaging markers computed from breast density distribution, textural features of parenchymal patterns, and parenchymal enhancement patterns are promising to build AI-based models to predict breast cancer risk. Study results have demonstrated that using image-based risk prediction models can perform superiorly to existing cancer risk prediction models that use epidemiological study data only. However, a majority of these state-of-the-art image-based risk models have not been tested or used in clinical practice due to lack of diversity in the training set leading to a model with poor generalizability on data from different locations and different scanners. Thus, these new image-based prediction models need to undergo vigorous and widespread prospective testing in future studies.

### Tumor Classification or Diagnosis

Due to the high rates of false-positive recalls and high number of benign biopsy results in current clinical practice using the existing imaging modalities, it is important to investigate new methods to help decrease the false positive recall and benign biopsy rates so that women are more willing to continue participating routine breast cancer screening. Over the past few decades, a variety of AI-based CADx schemes of different types of medical images have been developed aiming to differentiate between malignant and benign tumors more accurately to help radiologists decrease the false-positive recall rates in future clinical practice (**Table 3**).

In order to classify a detected tumor, many CADx schemes first segment the tumor or a ROI surrounding the suspicious area before computing image features. Some studies rely on semi-automated segmentation using prior knowledge of the tumor location marked by a radiologist as an initial seed, and other studies focus on fully automated segmentation. Dalmis et al. developed an AI-based CADx scheme for DCE-MRI that uses a semi-automated tumor segmentation technique prior to feature extraction. This is done by a multi-seed smart opening algorithm that first has the user identify a seed point and then a region growing algorithm is conducted followed by a morphological opening to segment out the tumor (81). El-Sokkary et al. recently investigate two new methods for the fully automated segmentation of the ROI from the whole breast mammogram prior to feature computation and classification. The first method segments the ROI using a Gaussian Mixture Model (GMM) and the second method uses a particle swarm optimization (PSO) algorithm. Twenty texture and shape features were then extracted from each ROI independently and used to train a non-linear SVM implemented with an RBF kernel. The accuracy of classifying malignant vs benign tumors using PSO-based segmentation and GMM-based segmentation prior to feature extraction was 89.5% and 87.5%, respectively (80).

To mirror the cognitive process of a radiologist in reading and interpreting bilateral and ipsilateral CC and MLO view mammograms of the left and right breasts simultaneously, researchers have developed and tested CAD schemes that integrate tumor image features with the corresponding features computed from the matched ROIs in other mammograms. For example, Li et al. conducted and reported a study in which image features were extracted from the segmented tumor region and the contralateral breast parenchyma; when these two feature sets were combined and used to train a Bayesian artificial neural network (BANN), there significantly improved tumor classification over the BANN trained using just features from the segmented tumor region (AUC = 0.84 vs 0.79, p=0.047) (89).

Identifying matched ROIs from different breasts is a difficult process. To avoid errors in tumor segmentation and image registration when identifying the matched ROIs in different images, researchers have investigated the feasibility of developing CAD schemes based on global image feature analysis of multiple images. For example, Tan et al. developed a CADx scheme using bilateral mammograms to classify screening mammography cases as malignant or benign. Ninety-two handcrafted features were extracted from each of the four view images and then concatenated into separate CC and MLO feature vectors, each containing the features from the left and right breast of the respective views. A multistage ANN was then trained where the first stage had two ANNs that were trained on either the CC feature vector or the MLO feature vector, and the second stage had a singular ANN that combine the classification scores output from both the prior ANNs and outputs a final score that estimates the likelihood of the case being malignant (88). To overcome the potential limitation of losing classification sensitivity from using the whole breast image, Heidari et al. developed a novel case-based CADx scheme that quantified the bilateral asymmetry between breasts using a tree structure-based analysis of the structural similarity index (SSIM). The left and right images are equally divided into four sub-blocks, the SSIM of each pair of two matched regions is calculated and a pair of the matched sub-blocks with the lowest SSIM among the original four pairs of sub-blocks is selected. The selected sub-blocks (one from left image and one from right image) are then divided into four small sub-blocks again to search for a new pair of matched sub-blocks with the smallest SSIM. This process is repeated six times. As a result, the six smallest SSIM features are extracted for each bilateral CC and MLO view images for each case. Then, three SVMs are trained and tested using a 5-fold cross validation method using the six SSIM features computed from the bilateral CC and MLO view images separately and the combined 12 SSIM features. Each SVM produces an outcome score indicating the likelihood of the case being malignant (90). The study demonstrates that using two bilateral images of MLO view yield significantly higher performance than using two bilateral CC view images (AUC = 0.75 ± 0.021 vs. 0.53 ± 0.026). However, fusion of SSIM features computed from both CC and MLO view images, SVM yields further increased classification accuracy with AUC = 0.84 ± 0.016.

Another popular method to eliminate the tumor segmentation step in CADx schemes is by using convolutional neural networks (CNN). CNNs can automatically learn hierarchical representations of the images directly from the image, eliminating the need for semi-automated or fully automated tumor segmentation and handcrafted feature selection. Due to the limitation of image dataset sizes in the medical imaging field, researchers have developed and trained shallow CNN models (127), which do not require as much training data as a deep CNN models. However, developing an architecture and training a CNN from scratch is still an extremely time-consuming process. Additionally, the robustness of studies using shallow CNNs is often questionable as they are trained on smaller dataset. Qiu et al. trained an eight-layer CNN to predict the likelihood of a mass being malignant, demonstrating that shallow CNNs can be trained fully on medical images (82). Yurttakal et al. trained a CNN with six convolutional blocks followed by five max pooling layers, a dropout layer, one fully connected layer, and a softmax layer to output a probability of malignancy of tumors detected on MR images. The accuracy of this system is 98.33% which outperformed many other studies of similar goals (83). The deeper a model is, the more complex representations can be learned, so the question of how deep a CNN must be to sufficiently capture features for a large classification task remains (128). However, training a deep CNN from scratch is not possible without a large diverse dataset which are not readily available in the medical imaging field.

**Figure 3:**
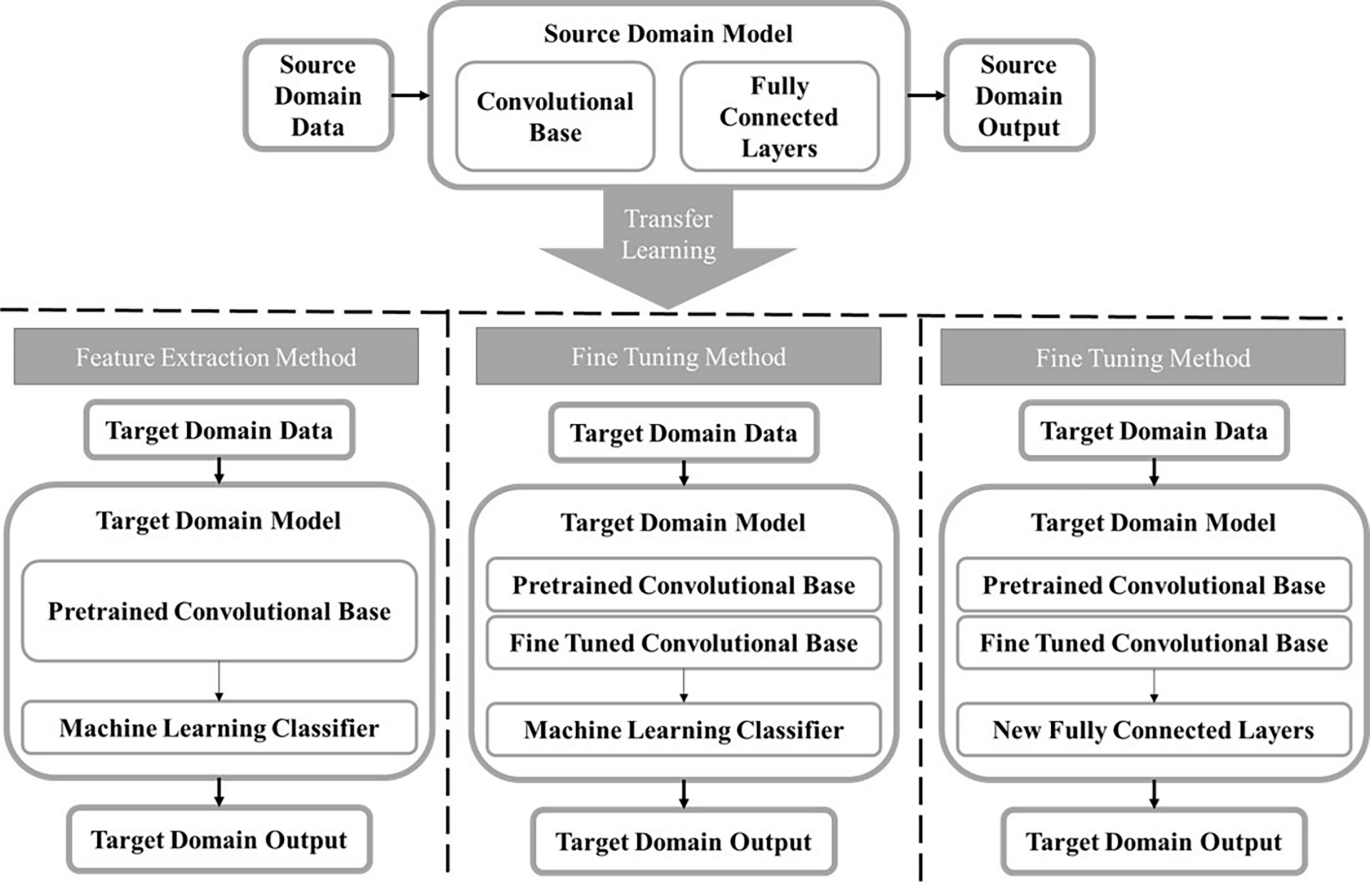
A block diagram displaying the transfer learning process. A model is trained in the source domain using a large diverse dataset. The information learned by the model is transferred to the target domain and used on a new task. The two main methods for transfer learning are feature extraction and fine tuning. For the feature extraction method, a feature map is extracted from the convolutional base taken from the source model and used to train a separate machine learning classifier. There are two ways to use transfer learning by fine tuning. The first is freezing the initial layers in the convolutional base from the source model and fine tuning the final layers using the target domain dataset then training a separate classifier. The second method does the same, except instead of training a new machine learning classifier, new fully connected layers will be added and trained using the target domain data.

By recognizing the limitation of shallow CNN models, transfer learning has emerged as a solution to lack of big data in medical imaging. In transfer learning, a CNN is trained in one domain and applied in a new target domain (129). This involves taking advantage of existing CNNs that have been pretrained on a large data set like ImageNet and repurposing them for a new task (130). There are two approaches to transfer learning (**Figure 3**), one is fine tuning where some layers of a pre-trained model are frozen while other layers will be trained using the target task dataset (131). The other is using a pre-trained network exactly as is to extract feature maps that will be used to train a separate ML model or classifier. The former is beneficial in that it will train the network to have some target specific features, but the latter is advantageous in that it is computationally inexpensive as it does not require any deep CNN training. In one study, Hassan et al. fine-tuned two existing deep CNNs, AlexNet and GoogleNet, that had been pretrained on the ImageNet database to classify tumors as malignant or benign using mammograms (84). The lower layers of each deep CNN were kept frozen, and the last layers of both networks were replaced to accommodate the two-class classification task and trained using the mammograms. Many different experiments were conducted to determine the most optimal hyperparameters for each deep CNN. The mammograms used in this study were a combination of images from four databases including the Curated Breast Imaging Subset of DDSM (CBIS-DDSM), the Mammographic Image Analysis Society (MIAS), INbreast, and mammogram images from the Egyptian National Cancer Institute (NCI), demonstrating the robustness of this fully automated CADx system. In another study, Mendel et al. used transfer learning as a feature extractor to compare the performance of a CADx model trained using DBT images and mammography images, independently. A radiologist placed a ROI around the tumor in corresponding the mammogram, DBT synthesized 2D image, and DBT key image which were then used as an input to the pre-trained VGG19 network. Features were extracted after each max-pooling layer. A stepwise feature selection method was used, and the most frequently selected features were used to train SVM models to predict the likelihood of malignancy. SVM model using DBT images yielded significantly higher classification accuracy than SVM model trained using mammograms, demonstrating that the features extracted from the DBT images may carry more clinically relevant tumor classification information than mammograms (85).

While deep CNN based models have seen tremendous success, traditional ML-based models that use handcrafted radiomic features benefit from prior knowledge of useful feature extraction methods making the handcrafted features more interpretable than automated features produced by deep learning models. Recently, fusion of traditional handcrafted features and deep learning-based features has been a hot topic and several studies report superior performance of the fusion approach over using either method alone. For example, Caballo et al. developed a CADx scheme for 3D breast computed tomography (bCT) images. The 3D mass classification problem was collapsed into a 2D classification problem by extracting nine 2D square boxes from each mass that mirror one of the nine symmetry planes of a 3D cube. The developed CADx scheme was then designed to take nine-2D images as an input. A U-Net based CNN model was used to segment the tumor from each of the nine 2D images. Then, 1,354 radiomic features were extracted from each image patch. The architecture of the rest of the proposed CADx scheme had two branches that work in parallel. The first arm of the system was a multilayer perceptron (MLP) composed of four fully connected layers that takes the radiomic features as an input. The second arm of the system was a CNN that processes the 2D image patch as is, meaning without the U-Net segmentation of the mass. The results of the last fully connected layer of both arms of the system were concatenated and processed by two more fully connected layers before tumor classification result is produced. The proposed model yielded AUC = 0.947 that outperforms three radiologists with AUC ranging from 0.814 – 0.902. This study demonstrates the utility of combining handcrafted features and CNN generated features in a singular CADx scheme (86).

Last, since original deep learning (CNN) models have been pretrained on a natural image data set like ImageNet, the models have three input channels to accept color images, yet medical images are typically gray scale images that only occupy a single input channel of the deep learning model. Thus, some studies directly copy the original grayscale image into three channels, while other studies added additional images into the other two input channels (28). Antropova et al. conducted a study that developed a classification model that fuses radiomics and deep transfer learning generated image features using a mammogram dataset, a DCE-MRI dataset, and an US dataset (87). The mammograms and ultrasound images were stacked in three input channels and fed to a pretrained VGG19 model, while the DCE-MRI pre-contrast (t0), first time-point (t1), and post-contrast (t2) were stacked in three input channels to form the input image of another VGG19 model. The deep CNN based features were extracted after each max pooling layer, average pooled in the spatial dimension, and concatenated into a final CNN feature vector. A semi-automated tumor segmentation method was used to segment the suspicious tumors before radiomic feature extraction. The radiomic and deep CNN feature set were used to train non-linear SVM with an RBF kernel using 5-fold cross validation. To build the fusion classifier the outputs of each SVM were averaged. Classifiers trained using the fusion of the two types of features outperformed all classifiers that used either feature set alone, demonstrating that traditional radiomic features and features extracted from transfer learning may provide complimentary information that can increase the performance of CADx schemes to help radiologist better make decisions. In addition to developing this CADx scheme for three independent imaging modalities, this study also demonstrated that features extracted from each max pooling layer of a pretrained CNN outperformed features extracted from the fully connected layers. This is significant as authors claim this is the first study using a hierarchical deep feature extraction technique for CADx of breast tumor classification. Similarly, Moon et al. developed a CADx scheme using multiple US image representations to train multiple CNNs which were then combined using an ensemble method (91). Four different US image representations were used: an ROI surrounding the whole tumor and tumor boundary that was manually annotated by an expert, the segmented tumor region, the tumor shape image which is a binary mask of the segmented tumor region, and a fused RGB image of the three prior image types. Multiple CNNs were then trained on each of the four image types and the best models were combined *via* an ensemble method. All models were evaluated using one private and one public dataset involving 1,687 and 697 tumors, respectively. Results of this study further demonstrate that the more information used in the input image, the better the model performs. Future work to automate the segmentation steps will improve the robustness of this model.

The above studies demonstrate that tumor segmentation remains one of the most difficult challenges that traditional ML based CADx schemes encounter and a major hurdle to clinical implementation. The shift from manual to semi-automated to fully automated lesion segmentation has decreased the inherent bias associated with human intervention, but elimination of the segmentation step in its entirety through either feature extraction from whole breast images or CNNs will be more generalizable than models involving a segmentation step when a large and diverse image database is available. Additionally, there remains no consensus on whether conventional ML models or new CNN-based DL models are better for breast lesion diagnosis as both methods have unique strengths and limitations. However, fusion of the two types of models has been shown to produce the best results as meaning these models may provide complementary information.

### Prediction of tumor response to treatment

Monitoring response to treatment is one of the most crucial aspects of breast cancer treatment and management. This must be done continuously through a combination of physical examinations, imaging techniques, surgical interventions, and pathological analyses. Molecular subtyping of each cancer based on histopathology into either luminal A, luminal B, human epidermal growth factor 2 (HER2) enriched, and basal-like subtypes is an important first step before deciding on the optimal treatment plan as each group has shown different responses to treatments and has varying survival outcomes (132, 133). Discovery of additional molecular signatures such as presence or absence of Ki67, expression of estrogen receptors (ER) and progesterone receptor (PR), cyclin-dependent kinases (CDKs), PIK3CA mutation, and others has opened the door for new targeted therapies that aim to inhibit cancer growth rather than shrink solid tumors (134, 135).

Neoadjuvant chemotherapy (NACT) is often used as a first line treatment with the goal of decreasing the size of the tumor. Evaluation of the efficacy of NACT is traditionally done through clinical evaluation using the Response Evaluation Criteria in Solid Tumors (RECIST), a size-based guideline (136, 137). The goal of the RECIST criteria is to categorize the response as either complete response (CR), partial response (PR), progressive disease (PD), or stable disease (SD). However, changes in the size of tumors will often not be detectable until 6-8 weeks in the treatment course therefore patients may continue experiencing the toxicity affects from chemotherapy or radiation therapy while not actually treating the cancer (138). In addition, the invention of many molecularly targeted therapies may be successful without showing a decrease in the size of the tumors, other factors such as change in vasculature or molecular composition may be better indicators of treatment response (139). Immunohistochemical (IHC) analysis can also be conducted before and after therapies to uncover molecular signatures and information about the vascular density of the tumor microenvironment (140–142). However, IHC analysis is an invasive procedure that is limited by the heterogeneity of the tumor since the biopsy sample is not necessarily reflective of the entire tumor (140, 143). The heterogeneity of tumors is a major hallmark of cancer, yet it is difficult to capture in a clinical setting making it difficult to predict response to therapy without knowing the entire molecular composition of the tumor. The need for non-invasive imaging markers that can quickly and accurately predict response to therapies has never been greater.

In current clinical practice, breast MRI is the most accurate imaging modality for monitoring tumor response to treatment as confirmed by The American College of Radiology Imaging Network (ACRIN) 6657 study performed in combination with the multi-institutional Investigation of Serial Studies to Predict Your Therapeutic Response with Imaging And molecular Analysis (I-SPY TRIAL) (144). In these clinical trials, radiologists read MR images and predict tumor response to treatment based on RECIST guidelines. In order to predict tumor response or cancer prognosis more accurately and effectively, many researchers have tried to develop AI-based prediction models of breast MR images acquired before, during or post therapy to predict tumor response to chemotherapy at an early stage.

In one study, Giannini et al. extracted 27 texture features from pre-NACT MRI and trained a Bayesian classifier to predict pathological complete response (pCR) post-NACT (92). In another study, Michoux et al. extracted texture, kinetic, and BI-RADS features from pre-NACT MRI to try and differentiate between individuals who would have no response (NR) and those who had either a partial response (PR) or complete response (CR) (93). Predictive capabilities of the features were analyzed independently and in combination through supervised and unsupervised ML models. Results showed that texture and kinetic features helped differentiate responders vs. non-responders, but BI-RADS features did not significantly contribute to the differentiation.

Aghaei et al. reported two studies that identified two new imaging markers by training two ANN models using kinetic image features extracted from DCE-MRI acquired prior to NACT to predict complete response (CR) to NACT (94). In the first study, an existing CAD scheme was applied to segment tumors depicting on DCE-MRI. Thirty-nine contrast enhanced kinetic features were then extracted from five groups: the whole tumor area, the contrast-enhanced tumor area, the necrotic tumor area, the entire background parenchymal region of both breasts, and the absolute value of bilateral BPE between the left and right breast. Using a leave-one-case-out cross validation method embedded with a feature selection algorithm, the trained ANN yielded prediction performance with an AUC = 0.96 ± 0.03 when 10 kinetic features were used. When comparing some of the common MRI features between the CR and NR groups using DeLong’s Method, no significant differences were seen between the two groups which demonstrates that conventional MR features alone may not have enough discriminatory power to predict whether a patient will respond to NACT or not. This study demonstrates that extracting more complex MRI features will yield greater performance in predicting the likelihood of a patient responding to NACT. As with many CAD studies, inclusion of the segmentation step often limits the robustness of the scheme. Thus, Aghaei et al. conducted a follow-up study using an increased image dataset and a new scheme that only computes 10 global kinetic features from the whole breast volume including average enhancement value (EV), standard deviation (STD) of EV, skewness of EV, maximum EV, average EV of top 10%, average EV of 5%, bilateral average EV difference, bilateral STD EV difference, bilateral difference of average EV of top 10%, and bilateral difference of average EV of top 5% without tumor segmentation. Then, by using the same ANN training and testing method, the ANN trained using 4 features yielded an AUC = 0.83 ± 0.04. Three of these four features were computed to characterize the bilateral asymmetry between left and right breasts, highlighting the key role that breast asymmetry may play in predicting whether a patient will respond well to chemotherapy (95).

**Figure 4:**
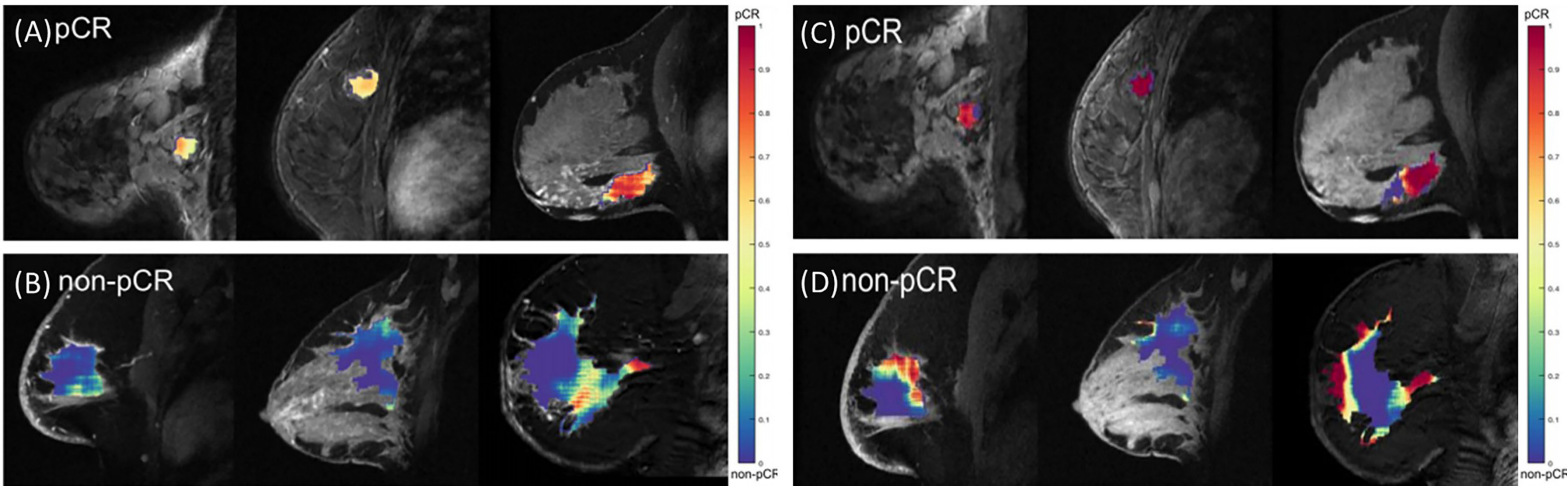
Illustration of heatmaps displaying the regions within a tumor that were used to predict the probability of pathological complete response. **(A, B)** show the results when using the CNNs trained on only the pre-contrast images. **(C, D)** show the results when using the CNN trained using a combination of pre-contrast and post-contrast images. **(A, C)** display cases that were correctly identified as pCR, while **(B, D)** are cases that were correctly identified as non-pCR. Modified from (96).

CNNs provide another tool that can overcome the limitations intrinsic to tumor segmentation steps. Ravichandran et al. used a CNN with six convolutional blocks trained over 30 epochs to extract features from pre-NACT DCE-MRI to predict the likelihood of a pathological CR (pCR) (96). This study looked at the pre-contrast and post-contrast images separately and together and found that the CNN performed best when using 3-channel images that contained the pre-contrast images in the red and green channel and the post-contrast images in the blue channel. The addition of clinical variables such as age, largest diameter, and hormone receptor status increased the AUC values from 0.77 to 0.85, demonstrating how the addition of AI can streamline imaging and clinical data into a single workflow for the increased prediction accuracy. Additionally, regions in the images that contain the most valuable information for predicting response to NACT can often be displayed in a heatmap (**Figure 4**). This may be an important step to reveal rationale of DL model prediction as few existing DL models are very interpretable which hinders their clinical translation.

Traditionally, pathological assessment of a representative tissue sample from the original tumor mass is used to identify the molecular subtype and develop a treatment plan. This is a sub-optimal technique as this representative tissue sample cannot capture the molecular composition of the whole tumor as cancer is often extremely heterogenous. Imaging modalities have the unique advantage of being able to capture information relating to an entire tumor which can help to overcome the limitations intrinsic to tissue biopsies. Additionally, the mechanism of many therapies is dependent on tumor vasculature which is not often probed before deciding on a treatment plan. Modalities that can image tumor vasculature such as DCE-MRI continue to be the most accurate and useful modalities in AI-based models for predicting response to treatment as valuable information pertaining to treatment response is contained in the tumor vasculature. Despite pre-clinical research progress, there are currently no image-based markers clinically used to predict response to any cancer therapies. Thus, more research efforts are needed to continue making progress to identify and validate robust image-based biomarkers that can predict response to therapy before the therapy is administered.

## Discussion – outlook and challenges

Breast cancer remains an extremely deadly disease with incidence on the rise. Early detection through routine screening exams remains the best method for reducing the mortality associated with the disease. However, the efficacy including both sensitivity and specificity of current breast screening must be improved. The increase in the number of breast imaging modalities coupled with a large amount of clinical, pathological, and genetic information has made it more difficult and time consuming for clinicians to digest all available information and make an accurate diagnosis and appropriate personalized treatment plan. Recent advances in radiomics and DL-based AI technology provide promising opportunities to extract more clinically relevant image features as well as to streamline many different types of diagnostic information to build novel decision-making support tools that aim to help clinicians make more accurate and robust cancer diagnosis and treatment decisions. In this review paper, we reviewed recent studies of developing AI-based models of breast images in three application realms.

In recent years, many “omics” topics including genomics, transcriptomics, proteomics, metabolomics, and others have attracted broad research interest in order to improve early diagnosis of breast cancer, better characterize the molecular biology of tumors, and establish an optimal personalized cancer treatment paradigm. However, these “omics” studies often require additionally invasive procedures and expensive tests generating high-throughput data that is difficult to do robust data analysis. Radiomics is advantageous in that it is non-invasive and low cost (because it only uses existing image data and does not require additional tests). Thus, the reported studies that directly apply radiomics concept and software to medical images has grown exponentially in recent years. In breast imaging, a large number of radiomics features can be extracted and computed such as from mammograms and DCE-MRI. Despite great research effort and progress, the association between radiomics and other “omics” is still not very clear and more in-depth research is needed. Thus, in this paper, we review several recent studies that investigated the relationship between radiomics features and the tumor microenvironment or tumor subtypes, which may provide researchers valuable references to continue in-depth research.

In addition, AI-based prediction models have expanded from the traditional task of detecting and diagnosing suspicious breast lesions in CAD schemes to much broader applications in breast cancer research. In this paper, we select and review application of AI-based prediction models to predict risk of having or developing breast cancer, the likelihood of the detected lesion being malignant, and cancer prognosis or response to treatment. These studies demonstrate that by applying either radiomics concepts through ML methods or deep transfer learning methods, clinically relevant image features can be extracted to build new quantitative image markers or prediction models for different breast cancer research tasks. If successful, the role of AI in breast cancer is paving the way for developing personalized medicine as detecting and diagnosing cancer are no longer driven by generic qualitative markers but now driven by quantitative patient specific data.

Despite the extensive research efforts dedicated to the development and testing of new AI-based models in the laboratory environment, very few of these studies or models have made into clinical practice. This can be attributed to several obstacles or challenges. First, currently, most of the studies reported in the literature trained AI-based models using small datasets (i.e., <500 images). Training a model using a small dataset often results in poor generalizability and poor performance due to unavoidable bias and model overfitting. Thus, one important obstacle is lack of large and high-quality image databases for many different application tasks. Although several breast image databases are publicly available including DDSM, INbreast, MIAS, and BCDR (87), these databases mainly contain easy cases and lack subtle cases, which substantially reduces the diversity and heterogeneity of these image databases. Many existing databases reported in previous research papers are also either obsolete (i.e., DDSM and MIAS used the digitized screen-film based mammograms) or have a lack of biopsy-approved ground-truth (i.e., INbreast). Thus, AI-models developed using these “easy” databases have lower performance in applying to real diverse images acquired in clinical practice. By recognizing such limitations or challenges, more research efforts continue to build better public image databases. For example, The Cancer Imaging Archive (TCIA) was created in 2011 with the aim of developing a large, de-identified, open-access archive of medical images from a wide variety of cancers and imaging modalities (145). New significant progress is expected in future studies to build this important infrastructure in help develop robust AI-based models in medical imaging field.

Second, medical images acquired using different machines made by different companies and different image acquisition or scanning protocols in different medical centers or hospitals may have different image characteristics (i.e., image contrast or contrast-to-noise ratio). CAD schemes or AI-models are often quite sensitive to the small variations of image characteristics due to the risk of overtraining. Thus, AI-models developed in this manner are not easily translatable to independent test images acquired by different imaging machines at different clinical sites. Compared to mammograms and MRI, developing AI-models of ultrasound images faces additional challenges because the quality of US images (particularly US images acquired using handheld US devices) heavily depends on the operators. Thus, establishment of TCIA allows researchers to train and validate their prediction models on imaging data acquired from other clinical sites to help researchers develop more accurate and robust models that can eventually be translated to the clinic. Additionally, developing and implementing image pre-processing algorithms to effectively standardize or normalize images acquired from different machines or clinic sites (146, 147) have also attracted research interest and effort, which may also need before AI-based models can be adopted on a widescale clinical level.

Third, another common limitation of traditional ML or radiomics based AI-based models is that they often require a lesion segmentation step prior to feature extraction. Whether lesion segmentation is done semi-automatically based on an initial seed or automatically without human intervention, accurate and robust segmentation of breast lesions from the highly heterogeneous background tissue remains difficult (148). The lesion segmentation error introduces uncertainty or bias to the model due to the variation of the computed image features and hinders the translation of the AI-based models to clinical applications. Recent attention to DL technology provides a way to overcome this limitation as the deep CNNs will extract features directly from the images themselves, bypassing the need for a lesion segmentation step. However, the lack of big and diverse datasets is a major challenge in developing robust DL-based AI models. Although transfer learning has emerged as a mainstream in the medical imaging field, its advantages and limitations are still under investigation. While there is a huge focus on using pre-trained CNNs as feature extractors as it is computationally inexpensive and generalizable since these models avoid having to train or re-train the CNN at different centers with different imaging parameters, fine tuning the models has showed better results (129). Additionally, no CNN-based transfer learning models have made it to clinical use since the models are still not robust as investigated in a recent comprehensive AI-model evaluation study (31). Therefore, more development and validation studies are needed to address and overcome this challenge.

Fourth, currently most AI-based models use a “black-box” type approach and lack explainability. As a result, it reduces the confidence or willingness of clinicians to consider or accept AI-generated prediction results (149). Understanding how an AI-based CAD scheme or prediction model can make reliable prediction is non-trivial to most individuals because it is very difficult to explain the clinical or physical meanings of the features automatically extracted by a CNN-based deep transfer learning model. Thus, developing explainable AI models in medical image analysis has emerged as a hot research topic (150). Among these efforts, visualization tools with interactive capability or functions have been developed that aim to show the user what regions in an image or image patterns (i.e., “heat maps”) contribute the most to the decision made by AI models (151, 152). In general, new explainable AI models must be able to provide sound interpretation of how the features extracted result in the output produced. Ideally this should be done in ways that directly tie to the medical condition in question. Since this is an emerging research field and important research direction, more research efforts should dedicate to extensive development of new technologies to make AI-based CAD schemes and/or prediction models more transparent, interpretable, and explainable before AI-based models or decision-making supporting tools can be fully accepted by the clinicians and then integrated into the clinical workflow.

Fifth, performance of AI-based models reported in the literature based on laboratory studies may not be directly applicable to clinical practice. For example, researchers have found that higher sensitivity of AI-based models may not help radiologists in reading and interpreting images in clinical practice. One previous observer performance study reported that radiologists failed to recognize correct prompts of CADe scheme in 71% of missed cancer cases due to higher false-positive prompts (153). By retrospectively analyzing a large cohort of clinical data before and after implementing CADe schemes in multiple community hospitals, one study reported that the current method of using CADe schemes in mammography reduced radiologists’ performance as seen by decreased specificity and positive predictive values (21). In order to overcome this issue, researchers have investigated several new approaches of using CADe schemes. One study reported that using an interactive prompt method to replace a conventional “second reader” prompt method significantly improves radiologists’ performance in detecting malignant masses from mammograms (154). However, this interactive prompting method has not been accepted in clinical practice. Thus, the lessons learned from CADe schemes used in clinical practice indicate that more research efforts are needed to investigate and develop new methods, including FDA clearance processes, to evaluate the potential clinical utility of all new AI-based models for many different clinical medical imaging applications (155).

In conclusion, besides CADe schemes that have been commercially available, advances in new technologies including data analysis of high throughput radiomics features and AI-based deep transfer learning have led to the development of large number of new CAD schemes or prediction models for different research tasks in breast cancer including prediction of cancer risk, likelihood of tumor being malignant, tumor subtypes or staging, tumor response to chemotherapies or radiation therapies, and patient progression-free survival (PFS) or overall survival (OS). However, before each of the new AI-based CAD schemes can be accepted in clinic practice, more work still needs to be done to overcome the remaining obstacles and validate its scientific rigor using large and diverse image databases acquired from multiple clinical sites. The overarching goal of this review paper is to provide readers with a better understanding of state-of-the-art status of developing new AI-based prediction models of breast images and the promising potential of using these models to help improve efficacy of breast cancer screening, diagnosis, and treatment. Additionally, by better understanding the remaining obstacles or challenges, we expect more progress and future breakthroughs will be made by continuing research efforts in the future.

## Author contributions

MJ writing of original manuscript preparation, revisions, and editing. WI, RF, XC writing, revisions, and editing. BZ. writing, revisions, editing, and funding acquisition All authors contributed to the article and approved the submitted version.

## Funding

This work was funded in part by the National Institutes of Health, USA, under grant number P20GM135009.

## Conflict of interest

The authors declare that the research was conducted in the absence of any commercial or financial relationships that could be construed as a potential conflict of interest.

## Publisher’s note

All claims expressed in this article are solely those of the authors and do not necessarily represent those of their affiliated organizations, or those of the publisher, the editors and the reviewers. Any product that may be evaluated in this article, or claim that may be made by its manufacturer, is not guaranteed or endorsed by the publisher.
